# Supplementary material for: Characterization of the Human Eccrine Sweat Proteome—A Focus on the Biological Variability of Individual Sweat Protein Profiles
Source: Int J Mol Sci. 2021 Oct 8;22(19):10871. doi: 10.3390/ijms221910871 (PMC8509809; doi:10.3390/ijms221910871)
Supplement: Supplementary file 1 [file ijms-22-10871-s001.zip › ijms-1413383-Supplementary.pdf]

Characterization of the human eccrine sweat proteome – a focus on the biological variability of individual sweat protein profiles

Bastien Burat[1ac], Audrey Reynaerts[2a], Dominique Baiwir[3], Maximilien Fléron[3], Gauthier Eppe[1], Teresinha Leal[2b], Gabriel Mazzucchelli[1bc]

[1] Mass Spectrometry Laboratory, MolSys Research Unit, Liège Université, Belgium

[2] Louvain Center for Toxicology and Applied Pharmacology (LTAP), Institut de Recherche Expérimentale et Clinique (IREC), Université Catholique de Louvain, Belgium

[3] GIGA Proteomics Facility, Liège Université, Belgium.

[a] equal contributors as first authors

[b] equal contributors as last authors

[c] corresponding authors

Correspondence: Bastien Burat (bastien.burat@uliege.be), Gabriel Mazzucchelli (gabriel.mazzucchelli@uliege.be)

Institut de chimie B6c, quartier Agora, Allée du six août, 11, B4000, Liège, Belgium

Phone: +32 (0) 4 366 34 11; fax: +32 (0) 4 366 43 87

Supplementary Information

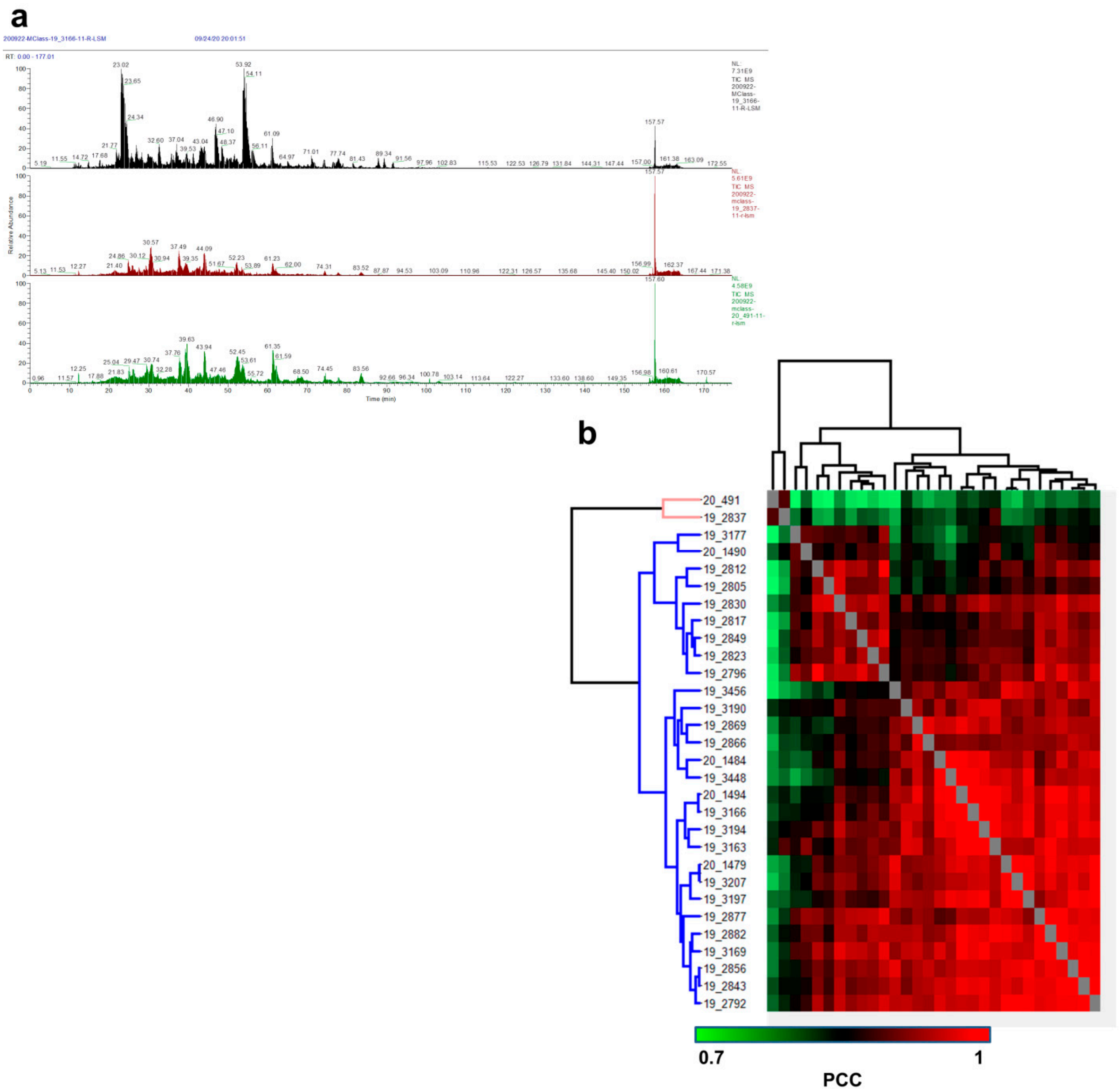

Supplementary Figure S1 – Discarded samples based on chromatogram discrepancy and poor correlation with other samples' protein profiles. a. TIC comparison. Reference TIC (black), TIC of discarded samples (red and green). b. Heat-map representation of 30 sweat protein profiles. Hierarchical clustering of Pearson's correlation coefficients generated using average Euclidian distance matrix.

Supplementary Table S23 – Quartile distribution of clinical data from hierarchically clustered samples. Color coding indicates value position in quartile-delimited distribution. Yellow: between minimum value and Q1, orange: between Q1 and Q2, bright red: between Q2 and Q3, dark red: between Q3 and maximum value. The cluster position column reports sample clustering from Figure 4b.

Clusters 1 and 7 grouped subjects with low collected volumes (low water loss) and ion amounts (low ion loss). Samples from cluster 7 would cluster apart due to higher protein masses and concentrations (higher protein loss).

Clusters 2, 3 and 5 grouped subjects with high collected volumes (high water loss). Samples from cluster 2 would cluster apart due to ion and protein secretion following the high water flow, resulting in high solute loss. Samples from cluster 3 would cluster apart because of ion and protein secretion not following the high water flow, resulting in low sweat concentration and solute loss. Then, the sample from cluster 5 would cluster apart because of a phenotype where ion and protein secretion alongside the high water flow, resulted in low potassium and protein concentration, on one hand, and high sodium chloride concentration, on the other hand.

Cluster 8 grouped subjects with discrepancies between right and left arm sweat volumes as well as a phenotype similar to cluster 5.

Cluster 6 grouped subjects with intermediate collected volumes (intermediate water loss) and high solute concentrations and amounts (high ion and protein losses).

Clusters 4 and 9 grouped subjects with unmatched sweat collected volumes and concentrations.
